# *Ganoderma lucidum* spore ethanol extract attenuates atherosclerosis by regulating lipid metabolism via upregulation of liver X receptor alpha

**DOI:** 10.1080/13880209.2020.1798471

**Published:** 2020-08-11

**Authors:** Peng Lai, Xu Cao, Qiao Xu, Yixin Liu, Rui Li, Ju Zhang, Meng Zhang

**Affiliations:** a School of Food and Bioengineering, Xihua University, Chengdu, China; b West China Hospital, Sichuan University, Chengdu, China; c School of Traditional Chinese Medicine, Chengdu University of TCM, Chengdu, China

**Keywords:** Triterpenic acid, reverse cholesterol transport, bile acid synthesis

## Abstract

**Context:**

*Ganoderma lucidum* (Leyss.ex Fr.) Karst (Ganodermataceae) is a fungus that has been used in traditional Chinese medicine.

**Objective:**

This is the first investigation of the lipid-lowering and anti-atherosclerotic effects of *Ganoderma lucidum* spore ethanol extract (EEG) in hyperlipidemic rabbits.

**Materials and methods:**

Fifty-four Japanese rabbits were randomly divided into six groups (*n* = 9): control, model, atorvastatin and three EEG groups (6, 24 and 96 mg/kg/day, p.o.). Control group was administered a normal diet and other groups were administered a high-fat diet to induce hyperlipidaemia and atherosclerosis for 14 weeks. During this time, lipid profiles were recorded; lipid testing and histopathological examination of aorta and liver were conducted. LXRα and its downstream genes expression in the liver and small intestine were examined. The effect of EEG on macrophage cholesterol efflux and ABCA1/G1 expression was observed under silenced LXRα expression.

**Results:**

EEG reduced serum cholesterol (20.33 ± 3.62 mmol/L vs 34.56 ± 8.27 mmol/L for the model group) and LDL-C, reduced the area of arterial plaques (24.8 ± 10% vs 53.9 ± 15.2% for the model group) and Intima/Medium thickness ratio, increased faecal bile acid content, upregulated LXRα, CYP7A1, ABCA1/G1, ABCG5/G8 expression in the liver, small intestine and macrophages. After silencing LXRα in macrophages, the ability of EEG to promote cholesterol efflux was inhibited.

**Discussion and conclusion:**

EEG exert lipid-lowering and anti-atherosclerotic effects via upregulating expression of LXRα and downstream genes associated with reverse cholesterol transport and metabolism. However, whether PPARα/γ are involved in the up-regulation of LXR expression by EEG remains to be elucidated.

## Introduction

Atherosclerosis (AS) is a common cardiovascular disease that leads to coronary heart disease, myocardial infarction, stroke and other peripheral vascular diseases that serve as serious threats to human health (Herrington et al. 2016). Its aetiology is complex and its risk factors include hypertension, diabetes, obesity, smoking and genetic factors. However, hyperlipidaemia, especially hypercholesterolaemia, is known as an independent risk factor for AS. Serum low-density lipoprotein (LDL-C) level is positively correlated with the incidence of AS. Many clinical studies have shown that lowering LDL-C can significantly reduce the incidence of AS and the associated cardiovascular and cerebrovascular events (Seo and Choi 2015). Reverse cholesterol transport that is mediated by high-density lipoprotein (HDL-C) can clear cholesterol deposited in the aorta and other sites, thereby reducing the incidence of AS. Hence, reducing HDL-C can also increase the incidence of AS (Hoekstra 2017). Due to the importance of cholesterol metabolism in the pathogenesis of AS, lipid-lowering drugs are the primary clinical treatment for AS. HMG-CoA reductase inhibitors (statins) and niacin drugs act by regulating cholesterol metabolism. These drugs are widely used clinically and have a definite therapeutic effect; however, they have some adverse effects. For example, statins can cause hepatotoxicity and rhabdomyotoxicity, and may even cause fatal rhabdomyolysis (Strandberg et al. 2014; Licata 2016). PCSK-9 inhibitors as monoclonal antibodies block PCSK-9 and reduce LDL-C, showing the effects of anti-atherosclerotic activities. However, the high cost of treatment limits its application on a larger scale (Schulman and Reed 2017). Moreover, little is known about the frequency with which adverse drug reactions (e.g., the risk of HCV and bacterial infection) to newly marketed medicines are experienced (Khademi et al. 2018). ANGPTL3 inhibitor is another promising new drug class for the treatment of hyperlipidaemia. Monoclonal antibodies and antisense nucleic acids are the two major types of ANGPTL3 inhibitors. Currently, these drugs are still not available on the market. Thus, due to the lack of post-marketing clinical data, safety and clinical efficacy are yet to be confirmed (Geladari et al. 2019). By considering the long-term nature of AS treatment, it is necessary to investigate new therapeutic targets and strategies, such as the development of medicinal foods that can be consumed for a long time, with the expectation to achieve lipid-lowering and anti-AS effects and adjunctive treatment while improving safety (Moore 2011).

Currently, the strategies to regulate cholesterol metabolism primarily aim to inhibit its biosynthesis, and its targets include HMG-CoA reductase and squalene synthase. Advances in basic research have provided new targets for lipid-lowering drugs, such as NPC1L1, which mediate cholesterol absorption in the small intestine. Its inhibitor, ezetimibe, is now available on the market and has achieved good clinical results. Transcription factors that regulate NPC1L1 protein expression include nuclear receptors such as peroxisome proliferator-activated receptors (PPARs) and liver X receptor (LXR) (Calpe-Berdiel et al. 2009; Lan et al. 2013; Balakumar and Mahadevan 2012) and they also regulate the ABC transporter superfamily, CYP7A1 and other targets closely associated with cholesterol metabolism. As a result, these nuclear receptors are increasingly valued as lipid-lowering and anti-AS targets with great potential and provide more flexible strategies for treatment and health care. However, other successful lipid-lowering drugs that act on nuclear receptors other than PPAR-α agonists (fibrates) are not currently available. Therefore, natural products with lipid-lowering and anti-AS effects that target receptors, such as LXR, have broad market prospects.

*Ganoderma lucidum* (Leyss.  ex  Fr.) Karst (Ganodermataceae) is a type of edible and medicinal fungus that protects the health of individuals and has been widely used for thousands of years in traditional Chinese medicine. *Ganoderma lucidum* contains polysaccharides, triterpenes, sterols and other biologically active compounds, which have immunomodulatory, anticancer and anti-pathogenic effects (Cor et al. 2018). *Ganoderma lucidum* spores contain active ingredients similar to those of *G. lucidum*, but the content of the major ingredients including polysaccharides and triterpenic acid is relatively higher and displays more prominent biological activities (Xie et al. 2013; Soccol et al. 2016). Existing studies have not systematically investigated the lipid-lowering and anti-AS effects of *G. lucidum* spores. However, our previous studies revealed that triterpenic acid and polysaccharides from some natural foods or herbs have significant lipid-lowering and anti-AS effects (Lai et al. 2011; Lai and Liu 2015). In the present study, we aimed to investigate the effect of *G. lucidum* spore ethanol extract (EEG) on a rabbit model of hyperlipidaemia and AS induced by high-fat diet and then attempt to reveal its possible mechanism of action.

## Materials and methods

### Preparation of *G. lucidum* spore ethanol extract

The identity of *G. lucidum* spore (purchased from Bazhong, Sichuan, China) was confirmed by the Natural Products Research Centre of Xihua University and a voucher specimen was deposited in Qinglongyi Agricultural Technology Co., Ltd. (Sample No. 20180128 M). Ultrasonic extraction of 5000 g of *G. lucidum* spore powder using 3 × 30 L ethanol was performed for 1 h. The crude extract was obtained by concentrating the solvent under reduced pressure. After re-dissolution in water, the crude extract was depigmented with activated charcoal and extracted with ethyl acetate. The experimental specimen *G. lucidum* spore ethanol extract (EEG) (15 g) was obtained by concentrating the organic phase under reduced pressure. EEG was chromatographed over C18 column with ethanol and determined by HPLC-UV. EEG was dissolved in normal saline at predetermined concentrations for *in vivo* and *in vitro* studies.

### Animal experiment design

A total of 54 male Japanese white rabbits (age, 3 months; initial body weight, 1.7–2.1 kg/rabbit) were provided by the Sichuan Provincial Experimental Animals Committee (Licence No.: SCXK 2013-14). Animals were routinely given special feed and drinking water at an ambient temperature of 18–26 °C and relative humidity of 30–70%. Animal experiments and husbandry were in compliance with the ‘Regulations on the Management of Laboratory Animals’ formulated by the State Scientific and Technological Commission.

All animals were randomly divided into 6 experimental groups based on their initial blood lipid values. Animals in the negative control group (Control) were given 50 g/day of normal feed, while the AS model group, positive control group (atorvastatin, Pfizer Inc, Dalian, China), and treatment groups (EEG-L, EEG-M, EEG-H) were given 50 g/day of high-fat diet (including 88% normal feed, 1% cholesterol, 3% lard, 8% egg yolk powder) (Zhang et al. 2002) as well as an intravenous injection of 200 mg/kg of 10% BSA (Sigma-Aldrich LLC, St. Louis, MO) at 4 weeks after the start of the experiment to promote atherosclerotic plaque formation (Rücker et al. 1988). Group design for the animal study was summarised in Table 1. The experiment lasted 14 weeks and body weight was measured every week. At the end of the 14th week, the animals were fasted for 12 h and then sacrificed. The liver, thoracic aorta, abdominal aorta and an ileal segment of the small intestine were rapidly harvested and washed twice with cold sterile normal saline. Approximately 1 cm fragment of the aortic arch near the proximal end and 1 g of liver tissue were collected and homogenised for lipid examination. Another small piece of liver and 1 cm of proximal aorta and abdominal aorta were fixed in 10% formalin solution for histopathological examination. The remaining tissue was frozen at −80 °C for Western blot examination.

**Table 1.** Experimental design.

Group	Number of animals	Diet	Oral dose (mg/kg/day)
Control	9	Normal	10 (N.S.)
Model	9	High-fat	10 (N.S.)
Atorvastatin	9	High-fat	2.5 (Atorvastatin)
EEG-L	9	High-fat	6 (EEG)
EEG-M	9	High-fat	24 (EEG)
EEG-H	9	High-fat	96 (EEG)

### Blood lipid examination

At the beginning of the experiment and at 4, 8 and 14 weeks after administration, fasting ear vein blood was collected. Thereafter, serum was separated by low-temperature centrifugation and total cholesterol (TC), low-density lipoprotein (LDL-C), high-density lipoprotein (HDL-C) and triglyceride (TG) levels were measured using an automated biochemical analyser (P800, ROCHE Ltd., Basel, Switzerland).

### Tissue lipid examination

Ten volumes of ice-cold normal saline were added to the aortic tissue for homogenisation. Subsequently, 10 mL of ice-cold normal saline was added to 1 g of liver tissue for homogenisation. The levels of lipids (TG and TC) in these tissue homogenates were measured using an automated biochemical analyser (P800, ROCHE Ltd., Basel, Switzerland).

### Faecal bile acid determination

The faeces of animals were collected on the last 7 days of the experiment and faecal total bile acid content was measured as described in the literature (Porter et al. 2003). Faecal samples (100 mg) were freeze-dried and refluxed for 120 min in a mixture of ethylene glycol-0.7 M potassium hydroxide to desorb bile acids, hydrolyse bile acid esters and deconjugate bile acids. After cooling, the solution was acidified and bile acids were extracted with diethyl ether. The ether extract was concentrated until dried. Thereafter, the residue was re-dissolved in methanol, and bile acid content was determined by enzymatic methods. Briefly, bile acid was oxidised to 3-ketosteroid with the presence of 3α-hydroxysteroid dehydrogenase (HSD) and Thio-NAD^+^, and Thio-NADH were generated. Then, 3-ketosteroid was reduced to bile acid by HSD and NADH. After multiple the redox cycling reactions, Thio-NADH was accumulated and amplified. Absorbance at 405 nm (Thio-NADH generated) was measured to obtain the content of total bile acid (TBA). The following calculation was performed:

(1) $$\mathrm{TBA}\;(\text{μM})=\frac{\text{change in sample absorbance }(\mathrm{Abs}/\min)}{\text{change in standard absorbance }(\mathrm{Abs}/\min)}\times \text{standard TBA concentration }(\mu M)$$

TBA standard solution: taurocholic acid, sodium salt (Sigma-Aldrich LLC, St. Louis, MO), 100 μM.

### Serum transaminase and creatine kinase measurement

Serum alanine aminotransferase (ALT), aspartate aminotransferase (AST) and creatine kinase (CK) levels were measured before administration and at 1, 2, 4, 8 and 14 weeks after administration. After the rabbits fasted for 12 h, 2 mL of blood was collected from the central ear artery to prepare 400 μL of serum. ALT, AST and CK levels were determined per the instructions provided by the kit manufacturers (Nanjing Jiancheng Bio-technology, Nanjing, Jiangsu, China).

### Histopathological examination

The abdominal aorta was fixed in 10% formalin solution for 72 h, rinsed with distilled water, washed once with 60% isopropyl alcohol, immersed in 0.5% Oil Red O solution for staining, washed and photographed. Plaque area was measured and calculated using Image-pro plus 5.2. The proximal aortic arch, thoracic aorta and liver were fixed, embedded in paraffin, sectioned and stained with HE. The pathological sections of each aorta were observed using a light microscope. The maximum thickness of the aortic intima and media was measured. The ratio of intimal and medial thickness was calculated as I/M = intimal thickness/medial thickness. Histological scoring of the liver was performed in a blinded manner and a score of 0–4 was assigned based on the proportion of cells with steatosis (0 points: <5%; 1 point: 5–30%; 2 points: 31–50%; 3 points: 51–75%; 4 points: >75%).

### Cell culture and ox-LDL uptake assay

The human monocyte THP-1 cell line was purchased from ATCC (Manassas, VA) and cultured in RPMI 1640 medium containing 10% FBS (Invitrogen Life Technologies, Carlsbad, CA), 100 U/mL penicillin and streptomycin. Cells were seeded in a Petri dish at a concentration of 6 × 10^5^ cells/mL and co-cultured with 100 nM phorbol ester (Sigma-Aldrich LLC, St. Louis, MO) for 72 h to induce differentiation into macrophages. Differentiated macrophages were seeded in 96-well plates, EEG was added to a final concentration of 0, 5, 10, 20 and 40 μg/mL, and cells were cultured at 37 °C, 5% CO_2_ for 24 h. The MTT assay (Sigma-Aldrich LLC, St. Louis, MO) was used to determine cell viability.

Ox-LDL was prepared as previously described (Lai and Liu 2014). Macrophages were seeded in 6-well plates, and 50 μg/mL ox-LDL and different concentrations of EEG (0, 5, 10 and 20 μg/mL) were co-cultured for 24 h to induce the formation of foam cells by macrophage for protein expression assays and Oil Red O staining. The foam cells were washed three times with PBS, fixed with 4% paraformaldehyde for 30 min, washed again with PBS and 60% isopropyl alcohol, immersed in 0.5% Oil Red O solution for 30 min, and finally washed three times with PBS and then observed under the microscope.

Small interfering RNA (siRNA) was transfected into macrophages using the Lipofectamine 3000 transfection reagent (Invitrogen Life Technologies, Carlsbad, CA, USA). Differentiated macrophages were co-cultured with human LXRα siRNA or negative control siRNA for 24 h following the manufacturer’s instructions. The siRNA for LXRα was designed online (http://rnaidesigner.thermofisher.com/rnaiexpress/). Negative control was provided by Invitrogen. The sequences were 5′-AUAACUGAAAUCCUUGAGGAAGGUG-3′ (siRNA for LXRα) and 5′-UUCUCCGAACGUGUCACGUTT-3′ (negative control). Cells were washed and co-cultured with 50 μg/mL ox-LDL and 20 μg/mL EEG for 24 h, and Oil Red O staining and protein expression assays were performed.

### Determination of CYP7A1 expression in liver

Microsomes were extracted from frozen livers as described previously (Guo et al. 2018). CYP7A1 protein concentration was determined using an ELISA kit (Abbkine, Wuhan, Hubei, China) for rabbit CYP7A1. Another sample of frozen liver tissue was used for total RNA extraction by homogenisation with Trizol reagent (Thermo Fisher Scientific, Waltham, MA). cDNA was synthesised using a reverse transcriptase kit (Qiagen, Valencia, CA). qPCR was performed using an ABI PRISM 7700 instrument (Thermo Fisher Scientific, Waltham, MA). The reaction system was 50 μL, with GAPDH as the internal reference, 20 ng cDNA template and 0.9 μmol/L concentration of forward and reverse primers (CYP7A1 primer sequence: Forward – GGAGAAGGCGAATGGGTGC, Reverse – GCACAGCCCAGATATGGAATC). CYP7A1 mRNA expression in liver tissue was compared to GAPDH mRNA expression.

### Western blot analysis of the expression of relevant proteins in tissues and cells

Frozen tissue was pulverised in liquid nitrogen and homogenised in RIPA buffer supplemented with protease inhibitor (Santa Cruz Biotechnology, Dallas, TX, USA). The homogenate was centrifuged at 20,000 *g* for 15 min at 4 °C, and the supernatant was collected. Protein was also extracted from macrophages as described above. The protein concentration of the supernatant was measured by the Bradford assay. Samples with the same amount of protein were separated by SDS-PAGE and transferred to a membrane. The membrane was sealed with skim milk and washed, followed by the addition of specific primary antibody (liver: LXRα, ABCA1/G1, ABCG5/8; small intestine: LXRα, ABCA1/G1, ABCG5/8; macrophage: LXRα, ABCA1/G1), with β-actin as the internal reference. The membrane was incubated overnight at 4 °C and washed. HRP-labelled secondary antibody was added and incubated for 1 h. Bands were visualised using enhanced chemiluminescence (ECL). All antibodies were purchased from Abcam (Cambridge, UK).

### Statistical analysis

All data were expressed as mean ± SD (SD shown by error bars). Student’s *t*-test was used for determining the differences between groups, and one-way ANOVA was used for analysing the differences between multiple groups. Liver histopathological score data were analysed by Mann–Whitney and Cruskal–Wallis non-parametric tests. *p* < 0.05 was considered statistically significant. SPSS software (18.0) was used for all analyses.

## Results

### HPLC profile of EEG

**Figure 1.**
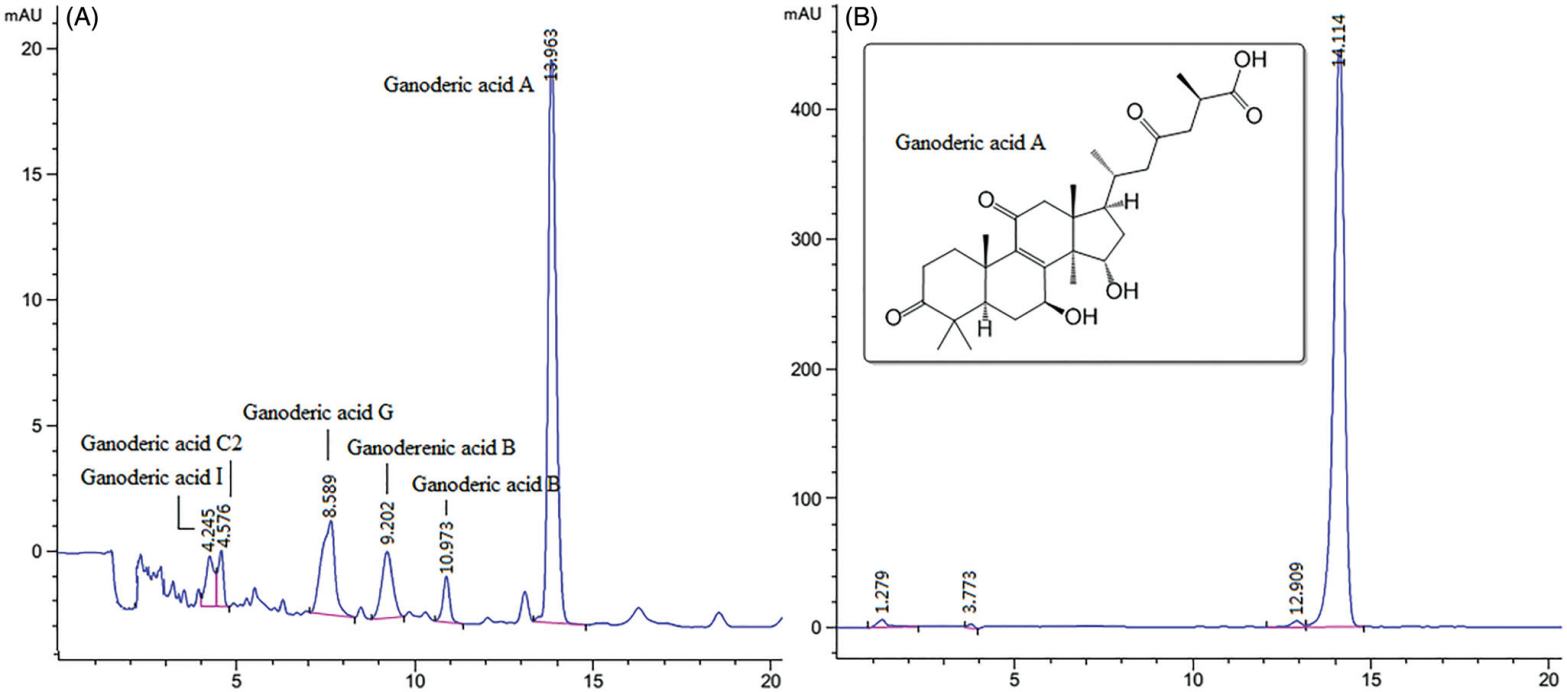
The HPLC chromatogram of EEG (A) and reference standards (B) analysed under the conditions: mobile phase, formic acid:acetonitrile:water (0.02:40:59.98, v/v), wavelength (254 nm), flow rate (1 mL/min), injection volume (20 μL), Column: Hypersil gold C18 (150 mm × 4.6 mm, 5 μm).

HPLC analysis of EEG and reference standards was carried out. The HPLC profile for EEG (Figure 1) revealed that the main triterpene content (about 60%) was ganoderic acid A (CAS No: 81907-62-2). Other triterpene contents in EEG were ganoderic acid G (CAS No: 98665-22-6), ganoderic acid B (CAS No: 81907-61-1), ganoderic acid C2 (CAS No: 98296-48-1), ganoderic acid I (CAS No: 98665-20-4) and ganoderenic acid B (CAS No: 100665-41-6). Triterpenoids accounted for about 90% of EEG.

### Effect of EEG on serum lipids

**Figure 2.**
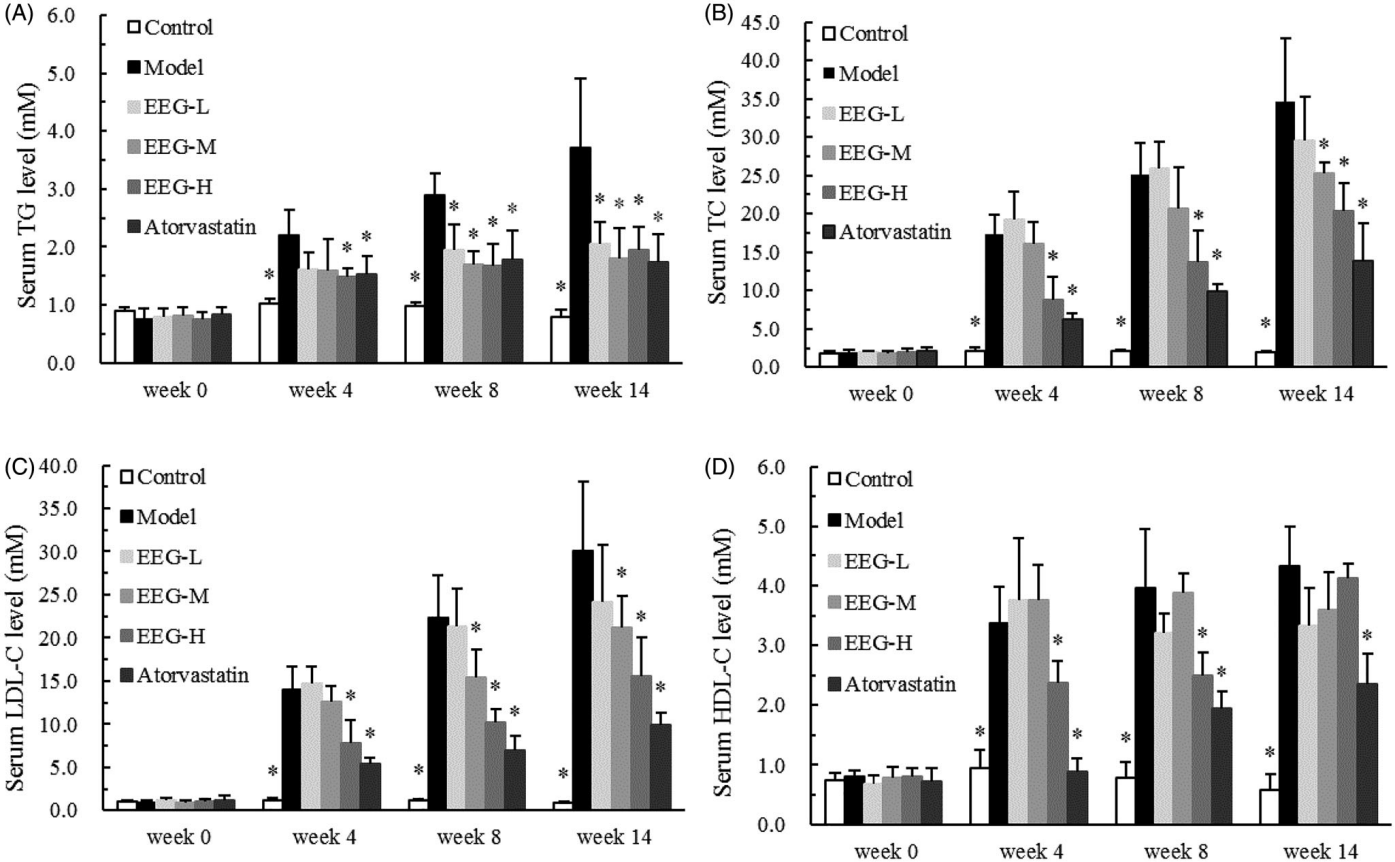
(A–D) EEG improved serum lipid levels in white rabbits with high-fat diet-induced hyperlipidaemia. Data were expressed as means ± SD (*n* = 9). *p < 0.05 vs Model group.

The effect of EEG on serum lipids in experimental rabbits is shown in Figure 2. High-fat diet significantly increased blood lipid levels in rabbits, with serum cholesterol levels increasing more than triglycerides. Subsequently, the hyperlipidaemia model was successfully established. Figure 2(A) shows that EEG significantly reduced serum TG levels in hyperlipidemic rabbits compared to the model group with an effect comparable to that of atorvastatin but without a significant dose-dependent relationship. Medium- and high-dose EEG (24 and 96 mg/kg/day) significantly reduced serum TC and LDL-C levels in hyperlipidemic rabbits and showed a good dose-dependent relationship (Figure 2(B,C)); however, they were not as effective as atorvastatin. Compared to the model group, serum TC levels in the EEG-M and EEG-H groups were, respectively, reduced by 7 and 49% at week 4, 18 and 46% at week 8 and 27 and 41% at week 14. The decrease in TC was primarily reflected in LDL-C. Compared to the model group, serum LDL-C levels in the EEG-M and EEG-H groups were respectively reduced by 10 and 44% at week 4, 31 and 54% at week 8 and 29 and 48% at week 14. In addition, EEG could elevate HDL-C compared to the model group (Figure 2(D)). Serum TC/HDL-C values of rabbits in the EEG-H group were significantly lower than those in the atorvastatin group (Table 2).

**Table 2.** Comparison of serum TC/HDL-C values at different time points in different experimental groups.

Group	TC/HDL-C ratio		
	Week 4	Week 8	Week 14
Control	2.50 ± 0.33	2.26 ± 0.26	2.63 ± 0.42
Model	5.13 ± 0.70	6.36 ± 1.06	8.09 ± 1.44
EEG-L	5.14 ± 0.70#	8.09 ± 1.68*#	8.83 ± 1.48#
EEG-M	4.30 ± 0.86#	5.31 ± 0.82	7.03 ± 1.53
EEG-H	3.63 ± 0.88*#	5.46 ± 0.80	4.93 ± 1.08*
Atorvastatin	6.69 ± 1.47*	5.07 ± 0.97*	5.90 ± 0.65*

Data were expressed as means ± S.D. (*n* = 9). #*p* < 0.05 vs Model group, *p < 0.05 vs Atorvastatin group.

### Effects of EEG on tissue lipids

**Figure 3.**
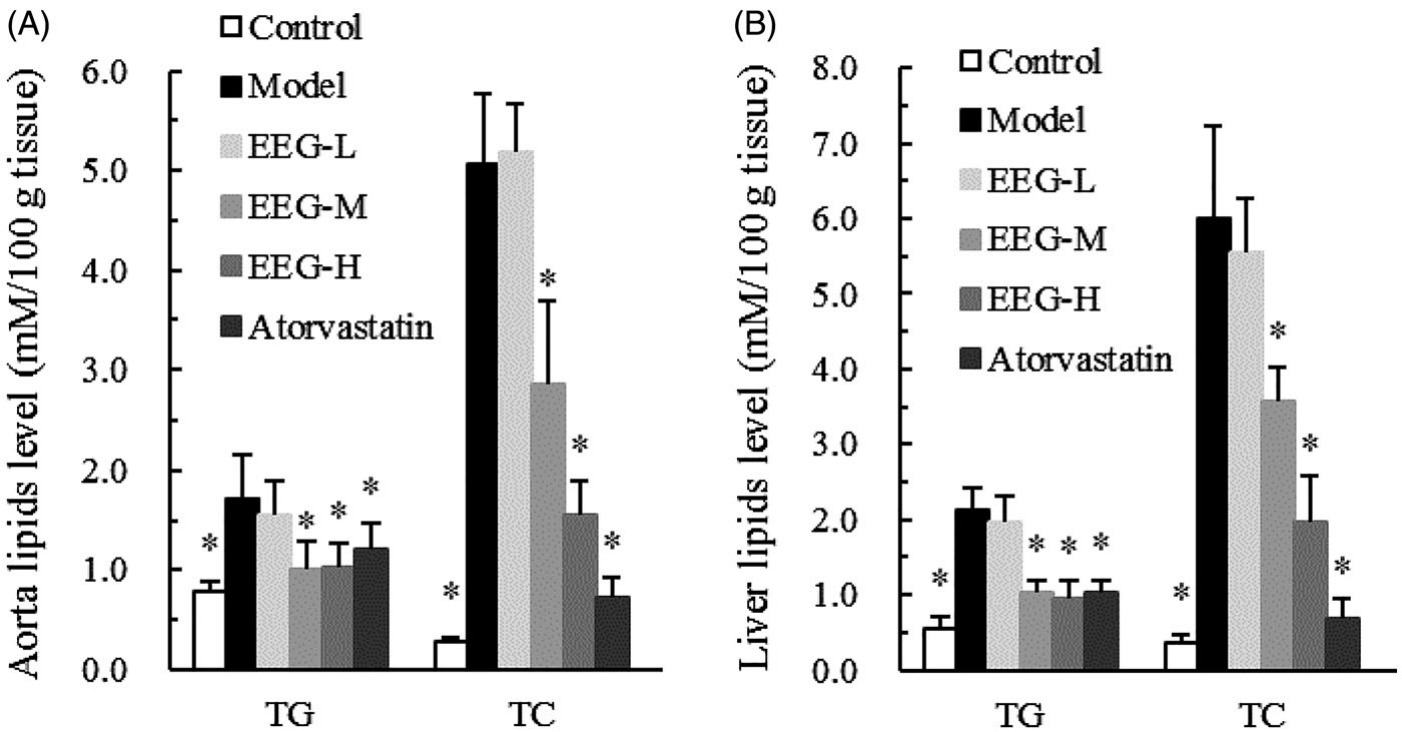
(A,B) EEG improved the aorta and liver lipid profiles in hyperlipidemic rabbits on a high-fat diet. Data were expressed as means ± SD (*n* = 9). *p < 0.05 vs Model group.

The effect of EEG on rabbit aorta and liver tissue lipids is shown in Figure 3. Compared to the model group, EEG treatment for 14 weeks significantly reduced TC levels in the aorta and liver tissue homogenates in a dose-dependent manner. In the aorta, TC levels in the EEG-M and EEG-H groups were 43 and 69% lower than those in the model group. In the liver tissue, TC levels in the EEG-M and EEG-H groups were 41 and 67% lower than those in the model group. In addition, TG content in the tissues was significantly reduced, but no dose-dependence was found.

### Effect of EEG on faecal bile acid content and CYP7A1 expression

**Figure 4.**
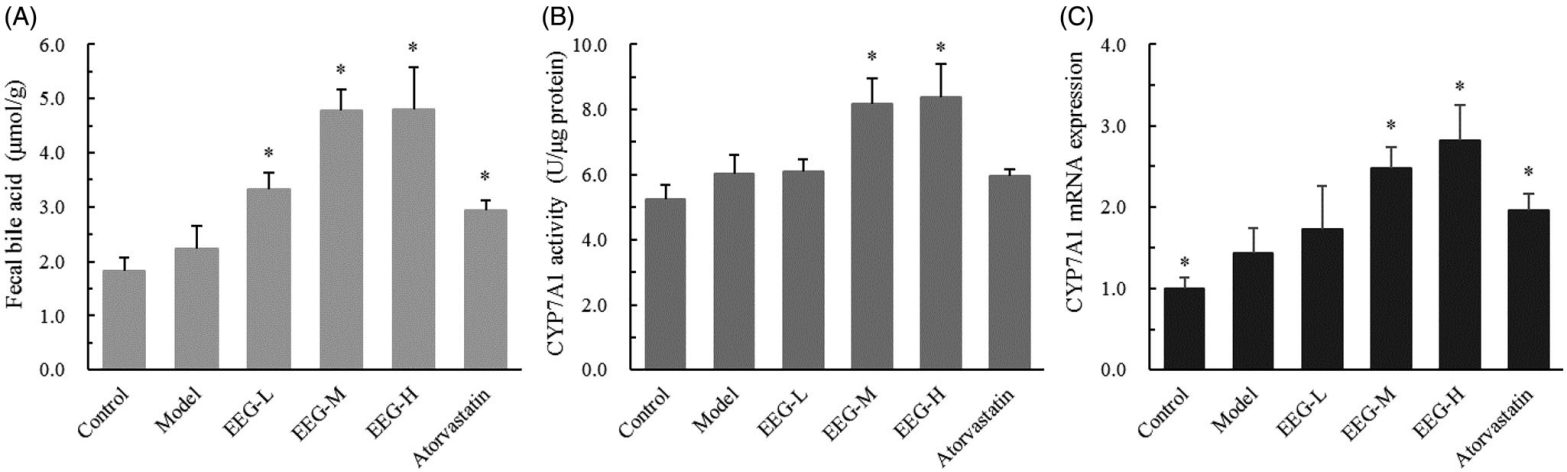
(A–C) EEG increases the expression and activity of CYP7A1 in the liver, thereby promoting bile acid synthesis. Data were expressed as means ± SD (*n* = 9). *p < 0.05 vs Model group.

To preliminary investigate the mechanism of EEG action, faecal bile acid content and CYP7A1 expression were determined in experimental animals. EEG treatment was found to significantly increase bile acid content in the faeces of the rabbit, with a magnitude that positively correlated with dose (EEG-L, EEG-M, EEG-H group increased by 32.7, 53.1 and 53.3%, respectively, compared to the model group, Figure 4(A)). Further studies showed that EEG increased the protein concentration of the bile acid synthesis rate-limiting enzyme, CYP7A1 and its mRNA expression in a dose-dependent manner (Figure 4(B,C)).

### Effect of EEG on serum transaminase and creatine kinase levels

**Figure 5.**
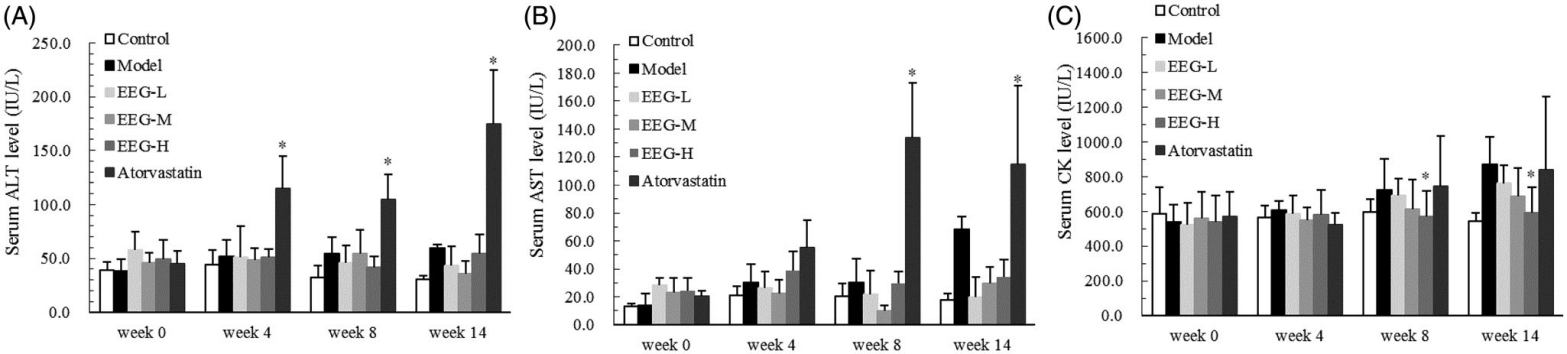
EEG has no significant effect on serum transaminase and creatine kinase levels. Data were expressed as means ± SD (*n* = 9). *p < 0.05 vs Control group (A and B) or Model group (C).

To determine whether the use of EEG in lipid-lowering therapy exerts the adverse effects of statins, the levels of serum transaminase and creatine kinase in experimental animals were measured. The results showed that there was no significant difference in ALT and AST levels between the EEG-treated groups and the model group, whereas ALT and AST were significantly increased in the atorvastatin group after 4 weeks of continuous treatment (Figure 5(A,B)). In addition, serum CK levels in the EEG-H group were significantly lower than in the model group after 8 weeks (Figure 5(C)).

### Effect of EEG on the area of aortic plaques and hepatic steatosis lesions

**Figure 6.**
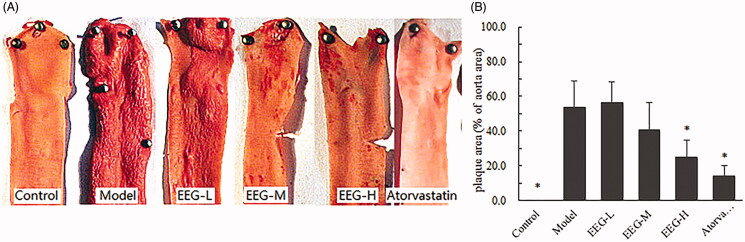
EEG significantly reduced the size of lipid plaques in the aorta of rabbits with hyperlipidaemia induced by high-fat diet. (A) Aortic lipid plaques after Oil Red O staining. Normal aorta was lighter and more uniform, whereas lipid plaques were stained scarlet by Oil Red O. (B) Aortic plaque area results were measured and calculated using Image-pro plus 5.2 (plaque area as a percentage of total area of aorta collected). Data were expressed as means ± SD. *p < 0.05 vs Model group.

**Figure 7.**
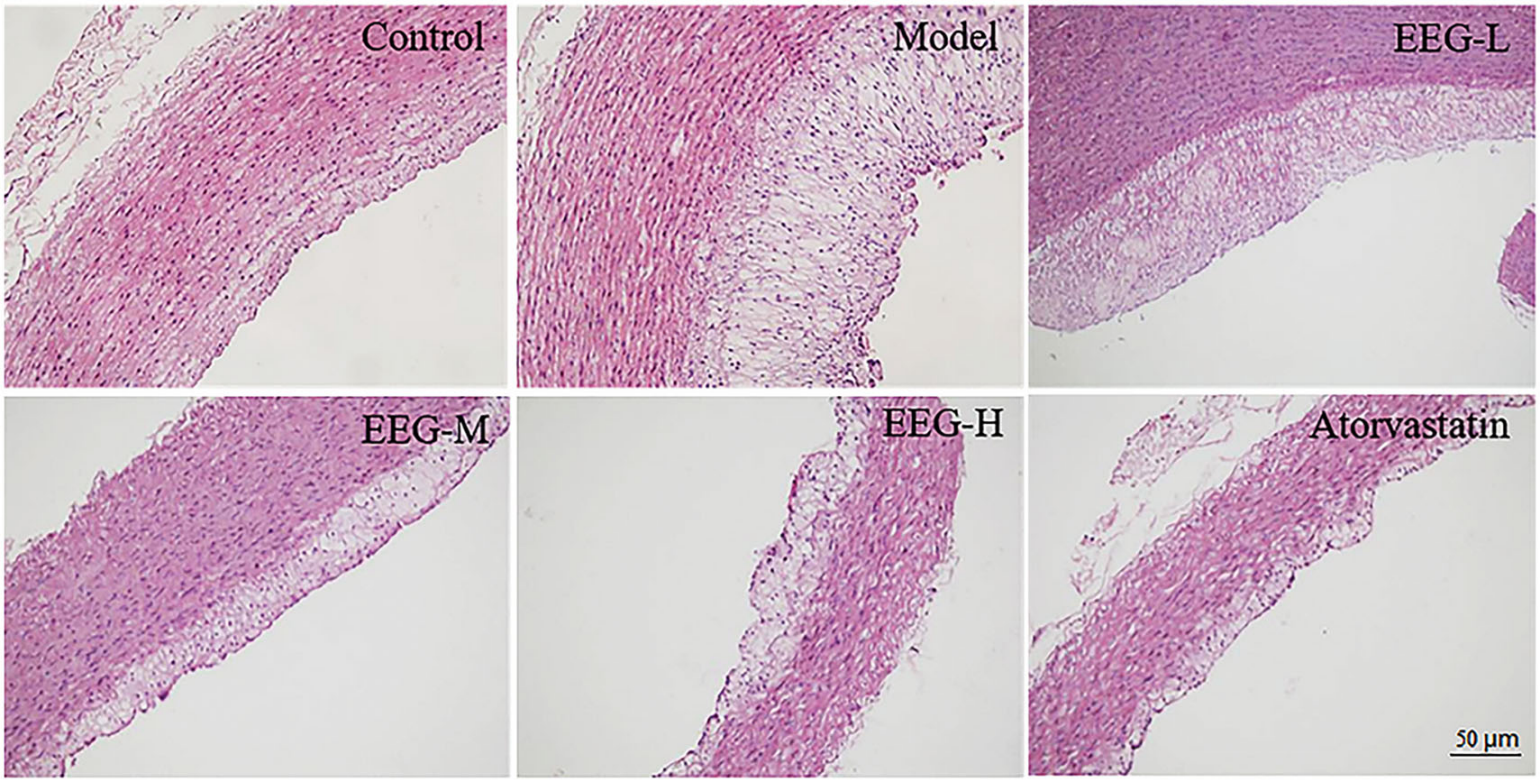
Representative results of HE staining of the aorta. EEG dose-dependently reduces the number of foam cells under the aortic endothelium of hyperlipidaemic rabbits.

Histopathological findings demonstrated the effect of EEG on the pathological processes of AS. The results of Oil Red O staining of the aorta are shown in Figure 6(A). Areas of aortic plaques in the EEG-H and atorvastatin group were significantly smaller than in the model group (Figure 6(B)). The condition of foam cells in the aorta was further examined by HE staining. The results revealed aggregation of many foam cells under the vascular endothelial cells in the model group and thickening of the intima of the arteries. EEG treatment reduced the number of foam cells and decreased the I/M ratio (Table 3) in a dose-dependent manner. To add, the number of foam cells observed in the EEG-H group was comparable to that in the atorvastatin group (Figure 7).

**Table 3.** Aortic intimal-medial thickness ratio and hepatic steatosis scores.

Groups	Aterial intima/medium thickness (I/M)	Hepatocyte steatosis (score)
Control	0.11 ± 0.08*	0.0 ± 0.0*
Model	1.06 ± 0.23	3.6 ± 0.5
EEG-L	0.84 ± 0.30	3.7 ± 0.5
EEG-M	0.36 ± 0.19*	2.5 ± 0.5*
EEG-H	0.30 ± 0.12*	0.8 ± 0.6*
Atorvastatin	0.26 ± 0.18*	#

Data were expressed as means ± SD (*n* = 9). *p < 0.05 vs Model group. #Atorvastatin group exhibited more severe chemical liver damage, which affected steatosis scores.

**Figure 8.**
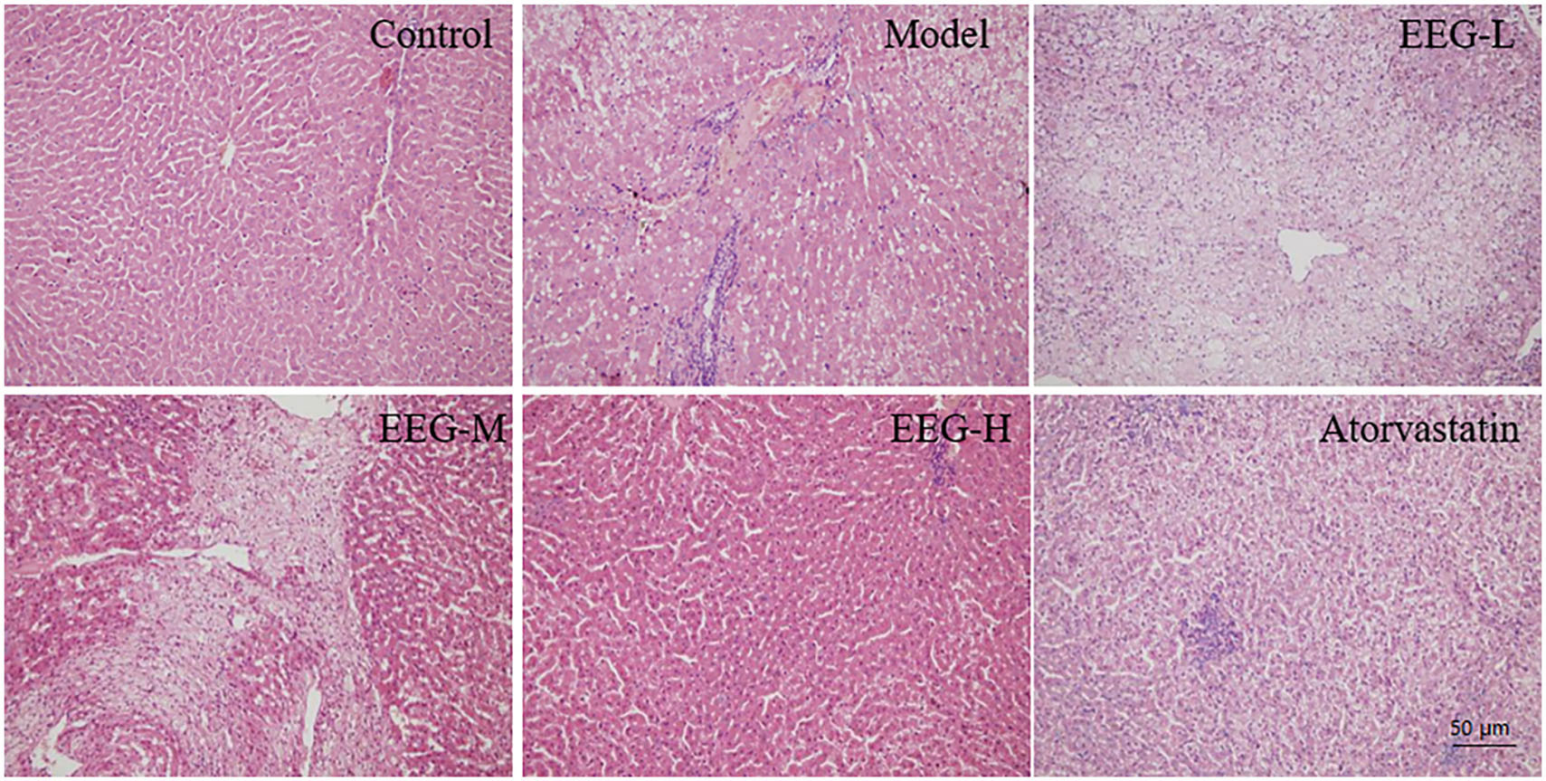
Representative results of HE staining of the liver. The effect of EEG on hepatic steatosis pathology in hyperlipidaemic rabbits showed that as dose increased, steatosis was reduced from diffuse (EEG-L) to focal (EEG-M) to nearly no steatosis in the EEG-H group. The atorvastatin group exhibited severe chemical liver damage.

HE staining of the liver tissue revealed the effect of EEG on hepatic steatosis in hyperlipidemic rabbits as shown in Figure 8. Compared to the control group, animals in the model group exhibited typical hepatic steatosis, and the cells in the liver tissue were arranged in a disorderly manner. Some hepatocytes were filled with many lipid droplets of different sizes and shapes, and infiltration of inflammatory cells could be seen in the hilar area. EEG significantly improved this pathological change, with a significant reduction in steatosis scores (Table 3) and only mild cellular oedema observed in the high-dose EEG group. The atorvastatin group showed typical pathological characteristics of chemical liver injury.

### Effect of EEG on foam cell formation

**Figure 9.**
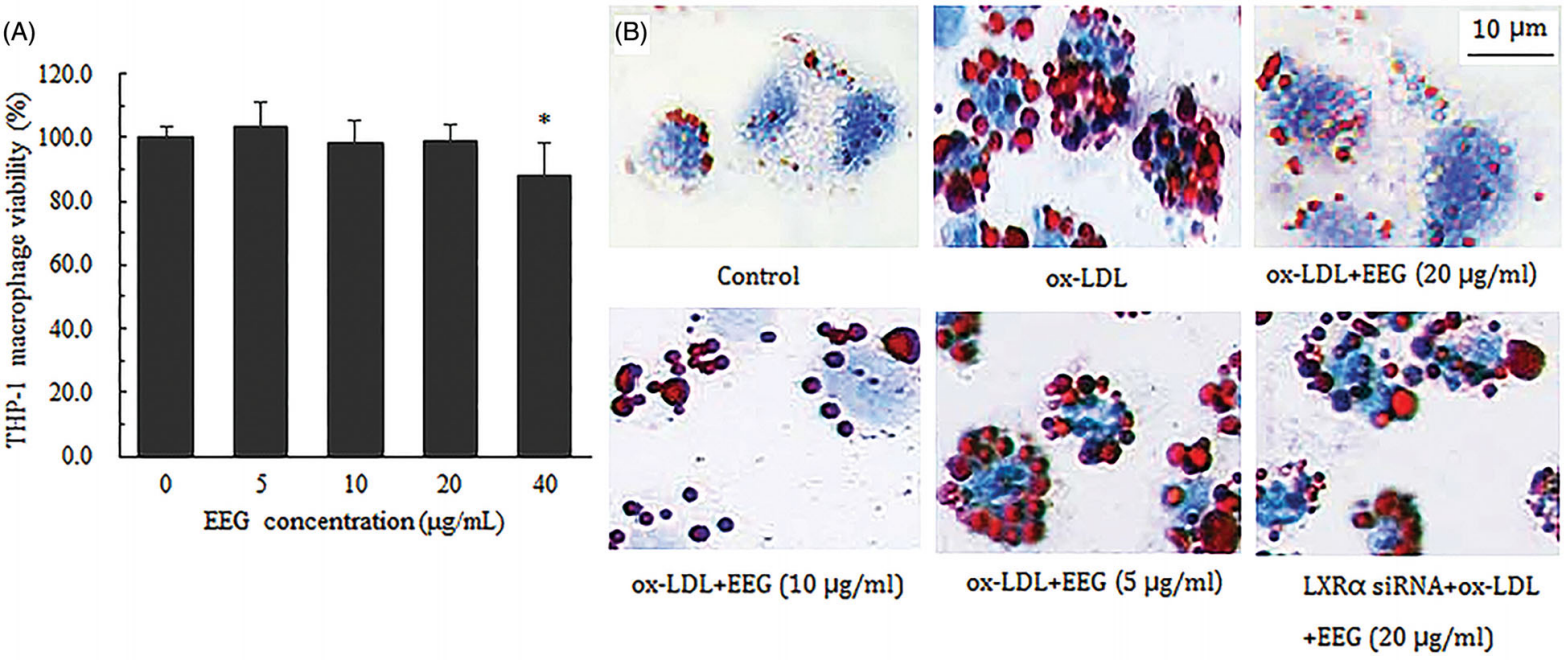
(A) Effect of different EEG concentrations on macrophage viability. (B) Results of Oil Red O staining of foam cells under different conditions. Data of MTT test were expressed as means ± S.D. *p < 0.05 vs Model group.

The concentration range for EEG *in vitro* assays was determined using a MTT assay. When the EEG concentration was 40 μg/mL, macrophage activity was significantly reduced (Figure 9(A)); hence, 20 μg/mL was selected as the maximum concentration.

In this study, Oil Red O staining was used to observe the role of different EEG concentrations on the formation of foam cells. The results showed that many cells stained red in the ox-LDL group compared to the blank control group, indicating that macrophages internalised many ox-LDL. After EEG treatment, the degree of staining decreased as the dose of EEG was increased. However, after the addition of LXRα siRNA, many cells were still stained red in the EEG group (20 μg/mL), indicating that the cells recovered ox-LDL uptake (Figure 9(B)).

### Effect of EEG on expression of relevant proteins in tissues and cells

**Figure 10.**
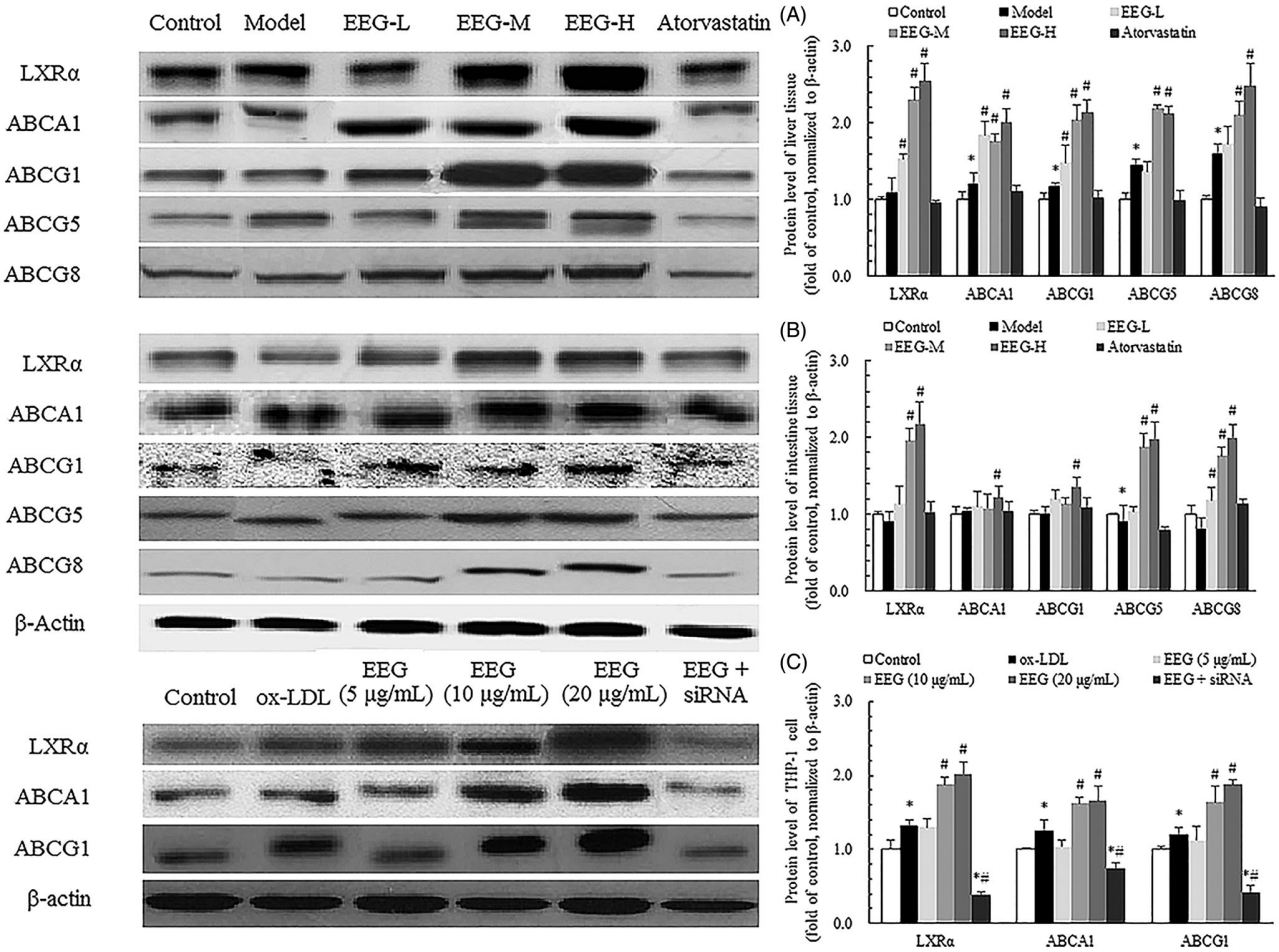
Effect of EEG on LXRα pathway in liver (A), intestine (B) tissues and macrophage (C). Western blotting was performed to determine expression of LXRα and its downstream molecules in the lysates of liver, intestine and macrophage. Each blot shows a representative result. The band density was measured and represents the expression of proteins. Data were expressed as means ± SD. *p < 0.05 vs Control group. #*p* < 0.05 vs Model or ox-LDL group.

To reveal the mechanism of action of EEG, the expression level of relevant proteins in tissues and cells was determined by Western blotting (Figure 10). The results showed that EEG significantly increased the expression of LXRα, ABCA1, ABCG1, ABCG5 and ABCG8 in the liver tissue compared to the model group. In particular, the levels of these proteins were increased by 57, 40, 46, 32 and 35%, respectively, in the EEG-H group.

In the small intestine, EEG significantly increased the expression of LXRα, ABCG5 and ABCG8 in a dose-dependent manner. Compared to the model group, the levels of the three proteins were increased by 59, 55 and 60%, respectively, in the EEG-H group.

In the *in vitro* assays, ox-LDL increased the expression of LXRα, ABCA1 and ABCG1 in THP-1 cells. After EEG treatment, the expression of these three proteins increased as the dose of EEG was increased. After the addition of LXRα siRNA, the expression of several proteins was significantly inhibited and the effect of EEG was offset.

## Discussion

In the context of the current consumer-based society, people consume large amounts of high-fat and high-calorie foods and drinks. Such intake has resulted in a rapid increase in the incidence of hyperlipidaemia. Hyperlipidaemia, especially hypercholesterolaemia, leads to AS, the underlying disease in cardiovascular events such as coronary heart disease and myocardial infarction. A major avenue for the prevention and treatment of AS is to emphasise the management of cholesterol metabolism (Aguilar and Fernandez 2014). Currently, the most commonly used cholesterol-lowering drugs in clinical practice are HMG-CoA reductase inhibitors (statins), which have significant lipid-lowering effects; however, they also cause a series of adverse reactions such as hepatotoxicity and rhabdomyotoxicity (Hippius et al. 2002; Karlson et al. 2013). Lipid-lowering therapy is a long-term process and the search for safe and effective lipid-lowering active natural products to ensure long-term use is an attractive strategy for preventing AS and cardiovascular and cerebrovascular diseases (Orekhov 2013; Liu and Huang 2016).

LXR is a member of the nuclear receptor superfamily. LXR is a transcription factor that regulates the expression of a series of transporters and metabolic enzymes closely associated with lipid metabolism, including ABCA1, ABCG1/G5/G8 and CYP7A1 (Fievet and Staels 2009). Numerous studies have shown that these active proteins play an important role in the reverse transport and circulation of cholesterol and can be used as targets for the development of cholesterol-lowering and AS prevention products (Crestani et al. 2004; Liao and McLachlan 2018; Yamanashi et al. 2018). LXR has two subtypes, LXRα and LXRβ, whose DNA homology is 80% (Calkin and Tontonoz 2010). LXRα is strongly expressed in the liver, small intestine, macrophages and other organs and is more closely associated with cholesterol metabolism, whereas LXRβ is more broadly expressed (Parikh et al. 2014).

*Ganoderma lucidum* is a special fungus used as a drug and food in China that exhibits a wide range of pharmacological activities (Shi et al. 2008; Yang et al. 2018; Cor et al. 2018). To our knowledge, the present study is the first to investigate the lipid-lowering and anti-AS effects of *G. lucidum* spore extracts as well as attempt to reveal its mechanism of action. The results of the study showed that prophylactic administration of EEG significantly improved serum and tissue (aorta and liver) lipid levels in rabbits with high-fat-diet-induced hyperlipidemias. Aligning with the lipid-lowering effect, EEG treatment reduced the area of aortic plaques and the number of foam cells in experimental animals. In addition, good control of hepatic steatosis was achieved, demonstrating that EEG has a good preventive effect on AS and non-alcoholic fatty liver disease (NAFLD) induced by hyperlipidaemia. The lipid-lowering effects of EEG differed from those of statins and are primarily characterised by a better regulatory effect on TC and LDL-C than on TG (the decrease in TG is small and no dose-dependent relationship is observed) and an obvious relative increase in the effect on HDL-C (TC/HDL-C reduction) is seen. HDL-C requires the involvement of ABC transporters (ABCA1, ABCG1 and so on) in the process of promoting efflux and reverse transport of cholesterol in foam cells and other cells (Tall 2008), which is an important indication for revealing the mechanism of action of EEG. The change in faecal bile acid content is another indication that EEG leads to an increase in bile acid content in animal faeces, suggesting an increased ability of the liver to metabolise cholesterol. Further studies showed that the expression of the bile acid synthesis rate-limiting enzyme CYP7A1 (7-α hydroxylase) was the cause of the increase in faecal bile acid content. Based on these results, EEG may exert its effects through LXRα. Activation of LXRα increases the expression of its target genes (*ABCA1*, *ABCG1*, *CYP7A1*) (Lobaccaro et al. 2001; Graham and Allen 2015), but such activation may also lead to physiological effects such as increased TG synthesis in the liver, which may be related to EEG with a greater effect on TG regulation than TC (Zhu and Li 2009).

To confirm this hypothesis, we examined the expression of LXRα and its target genes in the liver and small intestine of animals. As a result, EEG was found to significantly increase the expression of LXRα. Besides, a good dose-dependent relationship was found. Nonetheless, the expression of downstream genes of LXRα exhibited different results. In the liver, the expression of ABCA1/G1 and ABCG5/8 were generally consistent with that of LXRα (i.e., EEG treatment significantly increased the expression of these proteins in the liver). The liver is an important source of HDL and ABCA1 is responsible for the efflux of cholesterol to APOA1, which ultimately forms HDL. The increase in ABCA1 levels can enhance the ability of the liver to synthesise HDL (Phillips 2014a). ABCG1 assists HDL in its role in clearing cholesterol (Phillips 2014b). Increased ABCG5/8 expression can promote cholesterol secretion to bile acids (Othman et al. 2013), subsequently enhancing reverse cholesterol transport (RCT) and bile acid metabolism when coupled with increased CYP7A1 expression. EEG also increased ABCG5/8 expression in the small intestine, inhibiting the absorption of cholesterol in the small intestine and increasing the excretion of faecal sterols (Castro-Torres et al. 2015). The expression of these members of the ABC membrane protein family is regulated by LXR (Fievet and Staels 2009), but ABCA1 and ABCG1 expression levels in the small intestine did not significantly change; this might be caused by joint regulation by LXR and PPARγ (Jiang et al. 2017). The *in vitro* study results confirmed that LXRα is an essential protein for EEG function. During the formation of foam cells by macrophages, EEG also increased the expression of LXRα and its downstream genes, *ABCA1* and *ABCG1*, suggesting enhanced cholesterol efflux in macrophages. Such finding is consistent with aortic lipid content and histopathological findings. The expression of ABCA1 and ABCG1 in macrophages were significantly decreased after LXRα expression silencing. To add, the cholesterol efflux they mediate was also greatly reduced. This shows that the role of EEG is LXRα-dependent (i.e., EEG activates the LXRα nuclear receptor pathway by increasing protein expression, thereby increasing the expression of the downstream targets, ABCA1 and ABCG1 and promoting cholesterol efflux and RCT in macrophages). The cholesterol is transported back to the liver via the HDL pathway, which ultimately delays the formation of foam cells and the progression of AS.

Currently, the drug of choice for the clinical treatment of various types of hypercholesterolaemia and atherosclerosis is a statin that specifically inhibits β-hydroxy β-methylglutaryl-coenzyme A (HMG-CoA) reductase, the key rate-limiting enzyme in the process of cholesterol synthesis, blocking cholesterol synthesis and reducing TC and LDL-C levels to prevent AS. Long-term use of statins has significant adverse reactions, including hepatotoxicity and rhabdomyotoxicity, as confirmed in this study. After continuous atorvastatin treatment, serum ALT and AST levels were significantly increased in the experimental animals. Two animals in the atorvastatin group died in 11–14 weeks, and autopsy samples showed obvious chemical liver injury. After the end of the experiment, some liver samples of the atorvastatin group also revealed the signs of chemical liver injury. EEG did not exhibit these adverse reactions, and it could reduce the elevation of transaminases caused by hepatic steatosis. After high-dose EEG treatment, the liver of animals displayed no obvious signs of steatosis and serum CK level of the EEG-H group was significantly lower than that of the atorvastatin group. Such findings indicate that EEG had no obvious myotoxicity, and may have a better safety profile than atorvastatin, thereby providing new possibilities for lipid-lowering and anti-AS treatments.

## Conclusions

**Figure 11.**
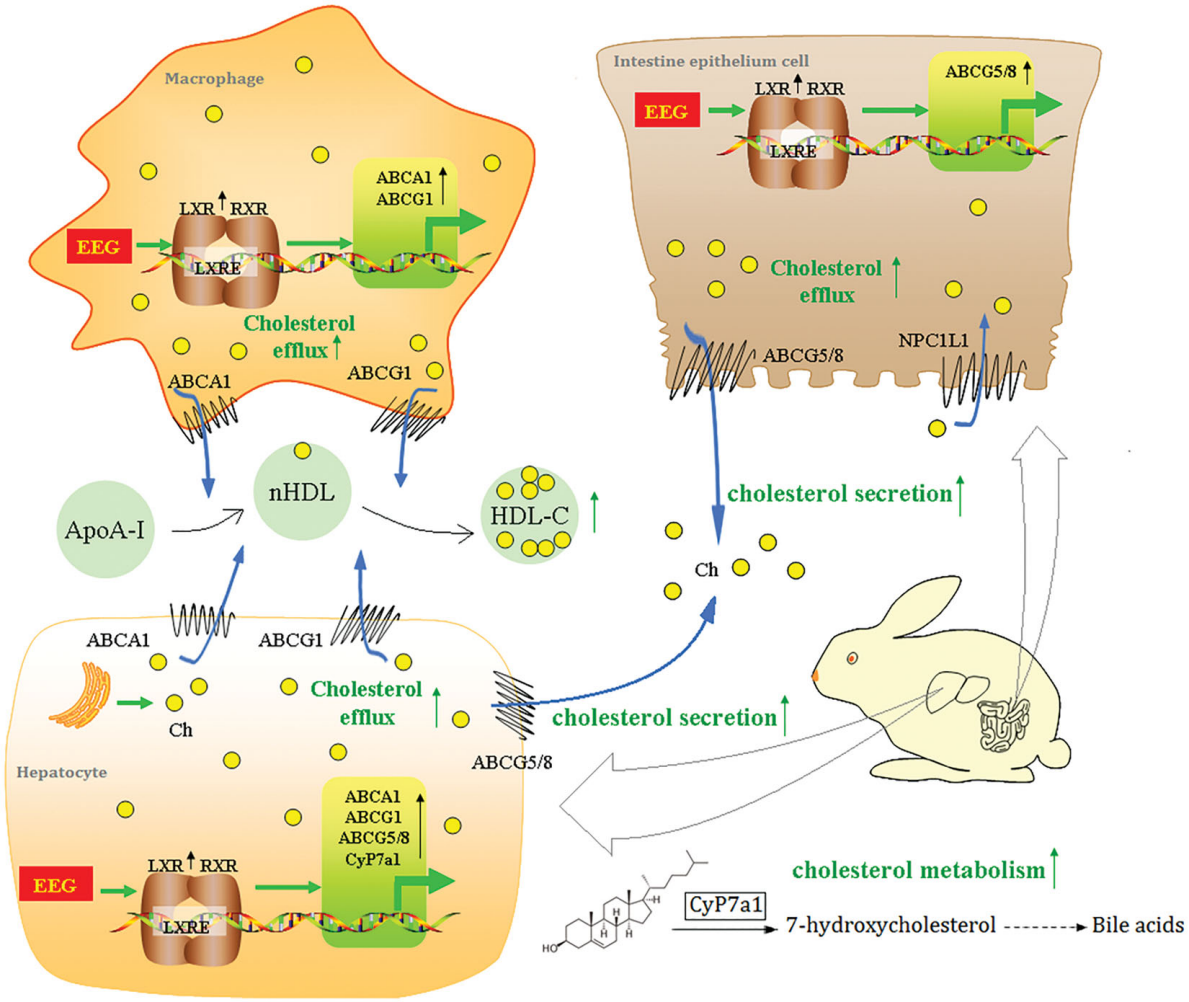
EEG attenuates atherosclerosis by regulating lipid metabolism via upregulation of LXRα.

In the present study, we investigated the lipid-lowering and anti-atherosclerosis effects of *G. lucidum* spore extracts and attempted to reveal its possible mechanism of action. EEG was found to significantly decrease serum TC and LDL-C levels and contribute to the increase in serum HDL-C levels, ultimately significantly delaying the pathogenesis of atherosclerosis. The mechanism of action of EEG may involve the upregulation of LXRα expression, and the subsequent increase in the expression of downstream target genes associated with cholesterol transport and metabolism (Figure 11). EEG is also a medicinal fungal extract that delays the progression of AS and exerts better safety than clinical drugs, thereby providing new strategies and directions for lipid-lowering treatments and atherosclerosis prevention. Besides, the potential value of traditional Chinese medicine and health care herbs such as *G. lucidum* is revealed, providing theoretical support for the development of future health care agents.
